# Family Caregivers as Advocates

**DOI:** 10.1093/geroni/igab046.246

**Published:** 2021-12-17

**Authors:** C Grace Whiting

**Affiliations:** National Alliance for Caregiving, Washington, District of Columbia, United States

## Abstract

This presentation discusses the growing influence of family caregiver advocacy and its prospects for impacting policy under the Biden administration, at both the federal and states levels. In particular, it will describe the National Alliance for Caregiving’s 50-state unified strategy for establishing the caregiver support infrastructure that is needed to coordinate efforts and to support caregivers across the nation and the lifespan. Historically, family caregivers have had difficulty acting as effective advocates, given the multiple roles they often play and their widely divergent interests, based on the varying needs of their care recipient and their divergent life circumstances. However, the Biden administration has indicated receptivity to caregiver issues, and the public has become increasingly aware of the caregiver role (in part, due to the pandemic), resulting in improved prospects for policy action at both the state and federal levels. This presentation reviews recent developments and discusses strategies for moving forward.

